# Single-Breath Counting Test Predicts Non-Invasive Respiratory Support Requirements in Patients with COVID-19 Pneumonia

**DOI:** 10.3390/jcm11010179

**Published:** 2021-12-29

**Authors:** Yaroslava Longhitano, Christian Zanza, Tatsiana Romenskaya, Angela Saviano, Tonia Persiano, Mirco Leo, Andrea Piccioni, Marta Betti, Antonio Maconi, Ivano Pindinello, Riccardo Boverio, Jordi Rello, Francesco Franceschi, Fabrizio Racca

**Affiliations:** 1 Department of Anesthesiology and Intensive Care, Azienda Ospedaliera SS Antonio e Biagio e Cesare Arrigo, 15121 Alessandria, Italy; lon.yaro@gmail.com (Y.L.); tatsiana_romenskaya@yahoo.it (T.R.); mirco.leo@outlook.com (M.L.); fracca@ospedale.al.it (F.R.); 2 Foundation of “Ospedale Alba-Bra Onlus”, 12060 Verduno, Italy; 3 Department of Emergency Medicine, Anesthesia and Critical Care Medicine, Michele and Pietro Ferrero Hospital, 12060 Verduno, Italy; 4 Department of Emergency Medicine, Polyclinic Agostino Gemelli University Hospital, 00168 Rome, Italy; saviange@libero.it (A.S.); andrea.piccioni@policlinicogemelli.it (A.P.); francesco.franceschi@unicatt.it (F.F.); 5 Department of Medicine and Emergency Medicine, Azienda Ospedaliera SS Antonio e Biagio e Cesare Arrigo, 15121 Alessandria, Italy; to.persiano@gmail.com (T.P.); rboverio@ospedale.al.it (R.B.); 6 Research Training Innovation Infrastructure, Research and Innovation Department, Azienda Ospedaliera SS Antonio e Biagio e Cesare Arrigo, 15121 Alessandria, Italy; marta.betti@ospedale.al.it (M.B.); amaconi@ospedale.al.it (A.M.); 7 Department of Sensory Organs, Sapienza University of Rome, 00161 Rome, Italy; ivano.pindinello@uniroma1.it; 8 Clinical Research/Epidemiology in Pneumonia & Sepsis (CRIPS), Vall d’Hebron Institute of Research (VHIR), 08035 Barcelona, Spain; jrello@crips.es; 9 Clinical Research in the ICU, CHU Nimes, Universite de Nimes-Montpellier, 34090 Montpellier, France

**Keywords:** COVID-19, pneumonia, single breath count, high-flow nasal cannula, continuous positive airway pressure (CPAP)

## Abstract

The use of non-invasive respiratory strategies (NIRS) is crucial to improve oxygenation in COVID-19 patients with hypoxemia refractory to conventional oxygen therapy. However, the absence of respiratory symptoms may delay the start of NIRS. The aim of this study was to determine whether a simple bedside test such as single-breath counting test (SBCT) can predict the need for NIRS in the 24 h following the access to Emergency Department (ED). We performed a prospective observational study on 120 patients with COVID-19 pneumonia. ROC curves were used to analyze factors which might predict NIRS requirement. We found that 36% of patients had normal respiratory rate and did not experience dyspnea at rest. 65% of study population required NIRS in the 24 h following the access to ED. NIRS-requiring group presented lower PaO_2_/FiO_2_ (235.09 vs. 299.02), SpO_2_/FiO_2_ ratio (357.83 vs. 431.07), PaCO_2_ (35.12 vs. 40.08), and SBCT (24.46 vs. 30.36) and showed higher incidence of dyspnea at rest (57.7% vs. 28.6%). Furthermore, SBCT predicted NIRS requirement even in the subgroup of patients without respiratory symptoms (AUC = 0.882, cut-off = 30). SBCT might be a valuable tool for bedside assessment of respiratory function in patients with COVID-19 pneumonia and might be considered as an early clinical sign of impending respiratory deterioration.

## 1. Introduction

The coronavirus disease (COVID-19) pandemia has stressed worldwide healthcare systems requiring a tremendous increase of the capacity of Emergency Departments (ED) to handle the sharp rise of patients in critical situation [1,2]. Pneumonia is the most frequent complication of infection, evolving in some patients to acute respiratory distress syndrome (ARDS) [2,3,4,5]. Hypoxemia was shown to be an independent prognostic factor for the severe form of COVID-19 and it was associated with in-hospital mortality [6,7]. Hypoxemia unresponsive to oxygen therapy suggests that gas exchange impairment is due to intrapulmonary shunt. In these patients the use of non-invasive respiratory strategies (NIRS), which include high-flow nasal cannula (HFNC) and non-invasive ventilation (NIV), allows to apply a positive end-expiratory pressure (PEEP) to the airways, which may reopen collapsed alveoli improving oxygenation and reducing intubation rate [8,9,10,11].

Early identification of critical patients requiring NIRS is essential to plan hospital resources and to prioritize monitoring efforts. Many patients with COVID-19-associated pneumonia showed severe dyspnea, increased respiratory rate with shallow breaths that precede oxygen saturation drops [12,13,14]. On the other hand, some patients presented “silent” hypoxemia without experiencing overt respiratory symptoms and this clinical presentation may cause a delay in the start of NIRS [15,16].

Previous works demonstrated that single-breath counting test (i.e., how far an individual can count in a normal speaking voice after a maximal effort inhalation) has good correlation with the standard measures of pulmonary function and it has been proposed as an indicator of respiratory compromised patients both in acute and in chronic setting [17,18,19,20].

The purpose of the study was to determine whether single-breath counting test (SBCT) may be useful in patients with COVID-19 pneumonia to predict the need for NIRS in the 24 h following the access to the Emergency Department (ED). Furthermore, we would like to define the cut-off limit of SBCT associated to progression of acute respiratory failure (ARF).

## 2. Materials and Methods

After the approval of our IRB with code n.: ASO.Rian.Gen.21/01 on 23 February 2021, we performed a prospective observational cohort study in two Italian hospitals. Patients with COVID-19 treated in the ED, who met criteria for pneumonia between 21 April and 22 July 2021 were enrolled. COVID-19 pneumonia diagnosis was defined by positive PCR in nasopharyngeal swab and by the presence of radiological or ultrasound patterns suggestive of the disease. We included in the study only patients treated in the ED for less than two hours. All patients provided written authorization for the use of their medical records for research. The institutional protocol of the coordinating center (protocol No. 0014676/21) was approved by the Ethics Committee on Clinical Investigations.

Exclusion criteria of the study included (1) patients already initiated on NIV or HFNC, (2) patients with severe hypoxemia (i.e, a PaO_2_/FiO_2_ < 150), (3) patients with severe dyspnea unable to speak in complete sentences, (4) uncooperative patients and (5) patients with a ‘do not resuscitate or intubate’ order. We evaluated patient’s ability to speak in complete sentences asking all patients to say name, surname, place and date of birth.

### 2.1. Protocol and Data Collection

Demographical characteristics (age, gender, body mass index), comorbidities and tobacco use were reported, SBTC, body temperature, pulse oximetry (SpO_2_), arterial blood gas (ABG) sampling, fraction of inspired oxygen (FiO_2_), respiratory rate (RR), heart rate (HR), presence of dyspnea at rest were collected upon inclusion into the study. In addition, blood tests were recorded.

Tachypnea in adults is defined as a respiratory rate greater than 20 breaths per minute. Dyspnea is defined as a subjective symptom described as an uncomfortable abnormal awareness of breathing, including a number of different sensations experienced by patients such as shortness of breath or inability to take a deep breath.

Investigators from each participating center were responsible for data collection; the protocol was explained and demonstrated during one specific educational meeting. The SBCT was performed by asking subjects to take a deep breath and count as far as possible in their normal voice at an approximate rate of two counts per second. Patients were instructed to stay in bed in the sitting position. We recorded two attempts, following a one-minute of rest between measurements.

Patients were followed up within the first 24 h of study inclusion and the level of respiratory support, such as low flow O_2_ therapy, HFNC, NIV, (i.e., continuous positive airway pressure—CPAP, or bi-level positive-pressure ventilation) or invasive mechanical ventilation (IMV) was reported.

We started conventional oxygen therapy when SpO_2_ was <92% on room air, using Venturi mask or nasal cannula and targeting SpO_2_ between 92–96% [8].

Patients with COVID-19 eligible for NIRS included subjects on conventional oxygen therapy having a PaO_2_/FiO_2_ ratio (P/F) < 250 or signs of increased work of breathing (i.e., RR > 30, accessory muscle use, abdominal paradox). In order to avoid that the decision to start NIRS was influenced by the SBCT value, this choice was left to the physician in charge who did not know the result of this test. The choice between CPAP, Bi-level Positive-Pressure ventilation or HFNC, as far as the decision to intubate and start invasive mechanical ventilation were based on clinical judgement of the physician in charge, who did not know the SBCT value.

### 2.2. Statistical Analysis

Quantitative variables were described with median and interquartile range (IQR), while qualitative variables were described with number and percentages. The statistical analysis was carried out using T student test for variables with normal distribution and as non-parametric test was used Mann-Whitney U test. The categorical data were tested with Chi2 test. The normal distribution was tested with Shapiro-Wilk test. The adjusted analysis was performed using penalized logistic regression models. The ROC curve was used to evaluate area under the curve (AUC) and cut-off. All tests were two sided and assumed a 5% significance level. Data analyses were performed using Addinsoft 2021 (XLSTAT Statistical and Data Analysis Solutions, New York, NY, USA).

## 3. Results

A total of 135 consecutive adults with COVID-19-associated pneumonia were treated during the study. We excluded 15 patients: ten for inability to perform SBCT, three for incompleteness of data and two for inability to obtain informed consent. Thus, the final number of patients enrolled in the study was 120 (mean age 66.9 ± 12.5 years old).

Demographic and clinical characteristics are shown in Table 1.

At inclusion in the study patients presented a mean P/F of 258.7 (±77.9). None of them had a P/F lower than 150. 68% were treated with conventional oxygen therapy. The rest of the patients breathed on room air. Many patients had dyspnea at rest (57 patients) or tachypnea (42 patients). 36% of all subjects (43 patients) had a normal respiratory rate (i.e., lower or equal to 20 breaths/min) and did not experience dyspnea at rest.

Patients were divided in two groups: the first one included 42 patients (35%) who didn’t required NIRS during next 24 h and the second group included 78 patients (65%) which required NIRS. Demographic characteristics were similar in the two groups (Table 1). The group which required NIRS presented significantly lower P/F, SpO_2_/FiO_2_ ratio, PaCO_2_ and SBCT scores and showed higher body temperature, higher incidence of dyspnea at rest and higher FiO_2_ (Table 1). In addition, NIRS-requiring group had higher values of LDH and ferritin, and had COVID-19 symptoms for more days.

**Figure 1:**
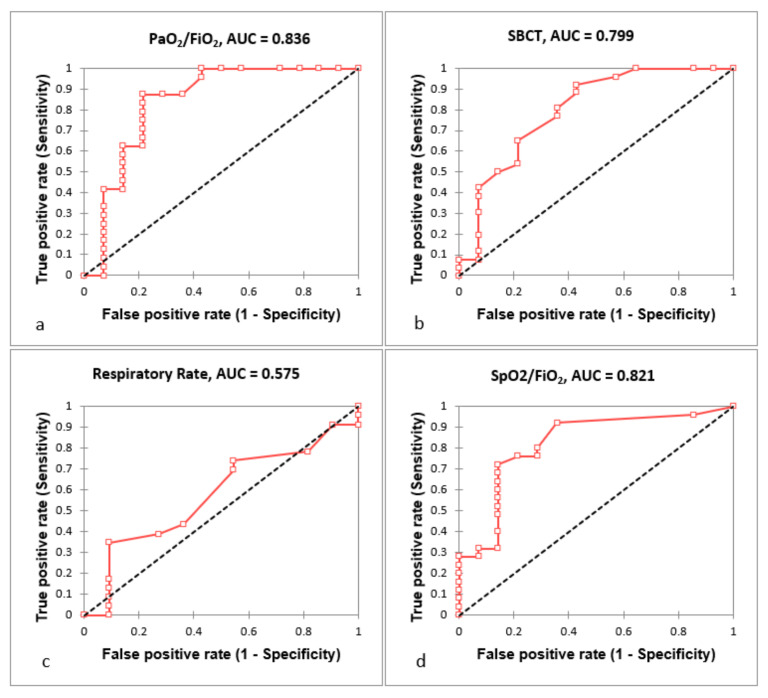
ROC curve analysis of PaO_2_/FiO_2_ (**a**), SBCT (**b**), Respiratory Rate (**c**) and SpO_2_/FiO_2_ (**d**) as predictors of NIRS requirement. Tests performed in all included patients. Abbreviations: NIRS, non-invasive respiratory strategies; SBCT, single-breath counting test.

The ROC curves were performed to evaluate AUC and calculate cut-off value for SBCT, respiratory rate, PaO_2_/FiO_2_ and SpO_2_/FiO_2_ in order to predict NIRS requirement in the next 24 h (Figure 1). The respective AUC, cut-off, sensitivity and specificity values are presented in Table 2 and clearly indicated that all parameters, except RR, predicted the need of NIRS.

In addition, we also evaluated the SBCT performance in predicting the use of NIRS analyzing subgroup of patients without respiratory signs or symptoms (Table 3).

**Figure 2:**
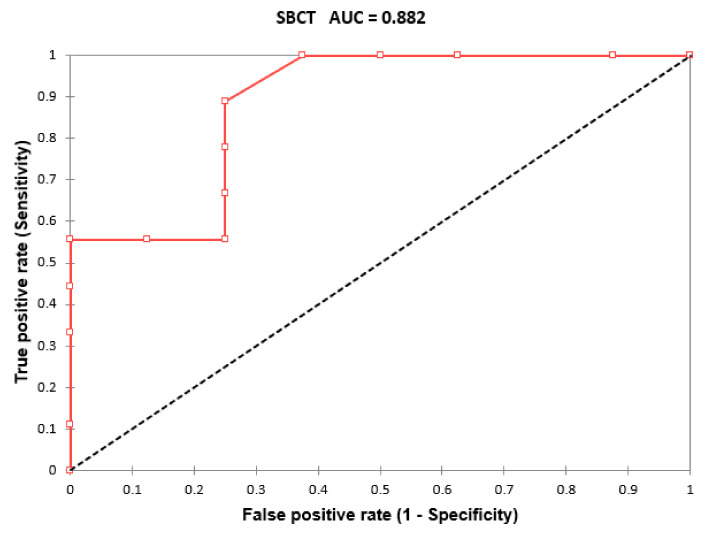
ROC curve analysis of SBCT performance in predicting NIRS requirement in the subgroup of patients with normal respiratory rate and absence of dyspnea at rest. Abbreviations: NIRS, non-invasive respiratory strategies; SBCT, single-breath counting test.

In particular, we analyzed separately patients without chronic respiratory disease, patients not receiving supplemental oxygen, subjects with normal respiratory rate (i.e., respiratory rate ≤ 20/min), without dyspnea at rest, with high P/F value (i.e., P/F > 280) or high SpO_2_/FiO_2_ ratio (i.e., SpO_2_/FiO_2_ ratio > 438). Both cut-off values were derived from ROC curves presented above. All subgroups except patients with SpO_2_/FiO_2_ ratio > 438 showed statistically significant difference in terms of SBTC between groups which required NIRS and group which didn’t required this therapy. Finally, we analyzed the group of patients without dyspnea at rest and with normal respiratory rate. In these population the SBCT was lower in patients requiring NIRS (25.11 ± 3.274 vs. 31.87 ± 4.52; *p* > 0.0001). The ROC curve analysis of this population highlighted the AUC equal to 0.882 [0.791–0.973], *p*-value < 0.0001 and cut-off values resulted to be 30 with sensitivity 0.889 and specificity 0.750 (Figure 2).

Among patients who used NIRS most subjects underwent CPAP with helmet (73 patients) and only five patients used HFNC. All patients included in the study were hospitalized or transferred to other hospitals within 24 h of accessing to ED.

## 4. Discussion

This study, focused on non-severely dyspneic and non-severely hypoxemic patients provides three major findings: (1) SBCT is valuable, replicable, easy to perform for bedside assessment of respiratory function in patients with COVID-19 pneumonia; (2) SBCT may predict the requirement of NIRS in the 24 h following the access to the ED, both in patients with obvious respiratory distress and in those with “silent” hypoxemia; (3) the cut-off limit of SBCT in patients without dyspnea at rest and with normal respiratory rate is 30, showing a good sensitivity and specificity.

COVID-19 pneumonia is characterized by substantial clinical heterogeneity. It spans from mild forms to severe forms with ARF and high intubation rates [21,22]. NIRS can decrease the need for intubation in patients with ARF [9,10,11,23]. On the other hand, in these patients a delay in the start of respiratory support might be particularly harmful. Consequently, early estimation of severity of COVID-19 pneumonia may help clinicians to decide patients’ allocation and interventions, in particular when an overwhelming load of admissions exceed the capacity of the ED. The presence of low SpO_2_ associated with dyspnea at rest is an excellent predictor of mechanical ventilation requirement [13,14]. However, many reports have described a subset of COVID-19 patients with hypoxemia showing no obvious respiratory difficulties [24,25,26]. In one of the first largest studies on the clinical characteristics of COVID-19, shortness of breath has been reported in only 18.7% of 1099 hospitalized patients, despite hypoxemia requiring supplemental oxygen was described in 41% of patients [27]. This phenomenon is referred as “silent” or “happy” hypoxemia and may result in a missed early recognition of evolving respiratory failure leading to delayed institution of respiratory support. In particular for these patients a bedside test predicting the need of NIRS should be highly advisable. For this purpose, the use of spirometry could be considered. Indeed, in the ED measurement of peak expiratory flow (PEF) is used to assess the severity of asthma [28] and forced vital capacity (FVC) is utilized to monitor patients with myasthenic crisis [29]. In addition, spirometry is used to evaluate patients who survive ARDS [30,31]. Thus, these measurements could also be useful for the assessment of respiratory function in COVID-19 patients. However, spirometry is not readily available in the ED. Moreover, it has the potential for aerosol generation during forced exhalation or coughing provoked by the maneuver [32]. As a consequence, we chose SBCT as bedside test to assess respiratory function in COVID-19 patients. Indeed, it correlates well with standard measures of pulmonary function both in acute and chronic setting [18,19]. It is already used in patients with asthma attack [18,19,20] or in patients with myasthenic crisis treated in the ED [29]. Elsheikh and coauthors showed that SBCT ≥ 25 suggests normal respiratory muscle function in patients with myasthenia gravis (MG) [20]. On the other hand, in the same patients a SBCT ≤ 20 has been proposed as an indication of respiratory compromise [33].

To our knowledge, this is the first study evaluating the use of SBCT in subjects with COVID-19 pneumonia. We found a good correlation between SBCT and the start of respiratory support in the next 24 h. In addition, SBCT appeared to perform consistently in discriminating between patients who required and who did not require NIRS. Good correlation was found also in the subgroup of subjects having a normal respiratory rate not experiencing dyspnea at rest. These results confirm that this measurement may be a useful tool for the assessment of patients with COVID-19 pneumonia who develop hypoxemia without showing first an obvious respiratory distress. In particular, in this subgroup a SBCT lower than 30 might identify patients requiring NIRS with good sensitivity and specificity. Even if we considered patients with P/F > 280 SBCT continued to be a good predictor of respiratory deterioration in the following 24 h.

All patients included in our study were at risk to devolve to severe hypoxemia and 65% of them were treated with NIRS in the 24 h following study inclusion. Of note, 36% of the enrolled patients had a normal respiratory rate and did not experiencing dyspnea at rest.

Our data confirms preceding studies which showed that in patients with COVID-19 pneumonia low P/F, low SpO_2_/FiO_2_ ratio, low PCO_2_ and the presence of dyspnea at rest predict the need of NIRS [12,13,14,34,35]. Of note, there is a slight discrepancy between PaO_2_/FiO_2_ and SpO_2_/FiO_2_ cut off, which predicts NIRS requirement (respectively 280 and 438). As far as the peculiar sigmoidal shape of the oxyhemoglobin dissociation curve is concerned, we can observe that in the higher range of arterial partial pressures of oxygen (PaO_2_) the upper part of the curve is flat. This feature prevents a significant decline in oxygen saturation when PaO_2_ starts to go down. Thus, SpO_2_ may underestimate an initial impairment of gas exchange [26].

This study has several limitations. The major limitation is that patients were only followed up for 24 h after study inclusion. Indeed, after the stabilization of clinical parameters, many patients were transferred to other hospitals due to the overwhelming inflow of COVID-19 patients. The second limitation is that patients during the 24 h of study period performed only one SBCT measurement. This limitation of the study protocol is aimed at reducing the exposure of investigators to the virus. Finally, we included in the study a relatively limited number of patients.

## 5. Conclusions

SBCT appears to be a promising tool in the early identification of patients with COVID-19 pneumonia who are at high risk of ARF requiring respiratory support. The simplicity of the SBCT makes this test appealing for rapid assessment of respiratory status also in patients with COVID-19 pneumonia treated in ED. More data from larger, prospective, multicenter studies are needed to confirm the efficacy of this test, possibly following SBCT trend for a longer time. In addition, SBCT could be evaluated in the assessment of COVID-19 outpatients to predict respiratory disfunction evolving to ARF, needing hospital admission.

## Figures and Tables

**Table 1:** Baseline characteristics of patients with COVID-19 pneumonia collected at inclusion into the study.

		NIRS Not Required (42)	NIRS Required (78)	*p*-Value
Age (years)		68.14 ± 13.62	66.15 ± 11.66	0.431
Gender	F	21 (50%)	27 (34.6%)	0.121
	M	21 (50%)	51 (65.4%)	
BMI (kg/m^2^)		25.67 ± 2.48	26.82 ± 5.79	0.449
Tobacco use		3 (7.1%)	3 (3.8%)	0.700
Comorbidities:				
Hypertension		30 (71.4%)	36 (46.1%)	0.007
Diabetes mellitus		3 (7.1%)	9 (11.5%)	0.433
Chronic kidney disease		6 (14.3%)	2 (2.5%)	0.017
Congestive heart failure		15 (35.7%)	12 (15.4%)	0.013
Coronary heart disease		12 (28.6%)	15 (19.2%)	0.248
Chronic respiratory disease		6 (14.3%)	12 (15.4%)	0.872
Clinical characteristics:				
Onset of COVID-19 symptoms (days)		5.3 ± 3.3	7.3 ± 3.8	0.01
Body Temperature (°C)		36.8 ±0.8	37.2± 0.9	0.038
Heart rate (bpm)		86.6 ± 7.4	85.8 ± 8.7	0.654
Respiratory rate (breaths/min)		21.5 ± 8.1	23.1 ± 7.7	0.314
Dyspnea at rest (Nº of patients)		12 (28.6%)	45 (57.7%)	0.02
SCBT		30.4 ± 6.9	24.5 ± 6.4	<0.0001
FiO_2_		0.2 ±0.02	0.3 ± 0.2	0.005
SpO_2_/FiO_2_		431.1 ± 39.1	357.8 ± 104.9	0.001
PaO_2_/FiO_2_		299.01 ± 95.1	235.1 ± 53.4	0.0001
PaCO_2_ (mmHg)		40.1 ± 6.7	35.1 ± 4.1	<0.0001
pH		7.4 ± 0.02	7.5 ± 0.06	0.0001
D-dimer (mcg/mL)		1.02 ± 0.8	0.9 ± 0.5	0.503
Ferritin (ng/mL)		489.9 ± 440.5	993.9 ± 910.6	0.003
LDH (U/L)		593.0 ± 107.2	698.9 ± 186.1	0.009

Abbreviations: NIRS, non-invasive respiratory strategies; BMI, body mass index; FiO_2_, fraction of inspired oxygen; SpO_2_, pulse oximetry; SBCT, single-breath counting test, LDH, Lactate dehydrogenase.

**Table 2:** ROC curves results of factors which might predict NIRS requirement. Tests performed in all included patients.

	AUC	Standard Error	Lower Bound (95%)	Upper Bound (95%)	Cut-Off	Sensitivity	Specificity	*p*-Value
SBCT	0.799	0.046	0.710	0.889	32	0.923	0.571	<0.0001
RR	0.575	0.059	0.459	0.691	28	0.348	0.909	0.206
PaO_2_/FiO_2_	0.836	0.057	0.725	0.948	280	0.875	0.786	<0.0001
SpO_2_/FiO_2_	0.821	0.052	0.720	0.923	438	0.720	0.857	<0.0001

Abbreviations: NIRS, non-invasive respiratory strategies; SBCT, single-breath counting test; RR, respiratory rate.

**Table 3:** ROC curve analysis of SBCT performance in predicting NIRS in different subgroups of patients.

	SBCT in Patients Not Requiring NIRS	SBCT in Patients Requiring NIRS	*p*-Value
Patients without chronic respiratory disease	30.7 ± 7.5	25.3 ± 5.1	<0.0001
Patients not needing supplemental oxygen therapy	30.1 ± 7.5	26.7 ± 3.0	0.007
Patients needing supplemental oxygen therapy	32.0 ± 0.0	20.8 ± 8.6	0.003
Patients without dyspnea at rest	31.5 ± 4.2	24.5 ± 3.2	<0.0001
Patients with PaO_2_/FiO_2_ > 280	29.7 ± 7.9	20.0 ± 6.6	0.0001
Patients with SpO_2_/FiO_2_ > 438	30.1 ± 7.9	27.3 ± 3.8	0.135
Patients with normal respiratory rate	30.2 ± 8.1	25.1 ± 3.2	0.0001
Patients with normal respiratory rate and absence of dyspnea at rest	31.9 ± 4.5	25.1 ± 3.3	<0.0001

Abbreviations: NIRS, non-invasive respiratory strategies; SBCT, single-breath counting test.

## Data Availability

Not applicable.
